# Performance of Machine Learning Suicide Risk Models in an American Indian Population

**DOI:** 10.1001/jamanetworkopen.2024.39269

**Published:** 2024-10-14

**Authors:** Emily E. Haroz, Paul Rebman, Novalene Goklish, Mitchell Garcia, Rose Suttle, Dominick Maggio, Eben Clattenburg, Joe Mega, Roy Adams

**Affiliations:** 1 Center for Indigenous Health, Johns Hopkins Bloomberg School of Public Health, Baltimore, Maryland; 2 Department of Mental Health, Johns Hopkins Bloomberg School of Public Health, Baltimore, Maryland; 3 Indian Health Service, US Department of Health and Human Services, Rockville, Maryland; 4 Department of Psychiatry, Johns Hopkins School of Medicine, Baltimore, Maryland

## Abstract

**Importance:**

Few suicide risk identification tools have been developed specifically for American Indian and Alaska Native populations, even though these populations face the starkest suicide-related inequities.

**Objective:**

To examine the accuracy of existing machine learning models in a majority American Indian population.

**Design, Setting, and Participants:**

This prognostic study used secondary data analysis of electronic health record data collected from January 1, 2017, to December 31, 2021. Existing models from the Mental Health Research Network (MHRN) and Vanderbilt University (VU) were fitted. Models were compared with an augmented screening indicator that included any previous attempt, recent suicidal ideation, or a recent positive suicide risk screen result. The comparison was based on the area under the receiver operating characteristic curve (AUROC). The study was performed in partnership with a tribe and local Indian Health Service (IHS) in the Southwest. All patients were 18 years or older with at least 1 encounter with the IHS unit during the study period. Data were analyzed between October 6, 2022, and July 29, 2024.

**Exposures:**

Suicide attempts or deaths within 90 days.

**Main Outcomes and Measures:**

Model performance was compared based on the ability to distinguish between those with a suicide attempt or death within 90 days of their last IHS visit with those without this outcome.

**Results:**

Of 16 835 patients (mean [SD] age, 40.0 [17.5] years; 8660 [51.4%] female; 14 251 [84.7%] American Indian), 324 patients (1.9%) had at least 1 suicide attempt, and 37 patients (0.2%) died by suicide. The MHRN model had an AUROC value of 0.81 (95% CI, 0.77-0.85) for 90-day suicide attempts, whereas the VU model had an AUROC value of 0.68 (95% CI, 0.64-0.72), and the augmented screening indicator had an AUROC value of 0.66 (95% CI, 0.63-0.70). Calibration was poor for both models but improved after recalibration.

**Conclusion and Relevance:**

This prognostic study found that existing risk identification models for suicide prevention held promise when applied to new contexts and performed better than relying on a combined indictor of a positive suicide risk screen result, history of attempt, and recent suicidal ideation.

## Key Points

**Question:**

Do published suicide risk machine learning models hold relevance for an American Indian population?

**Findings:**

This prognostic study of 16 835 adult patients that focused on testing the performance of existing published suicide risk models found that one model had an area under the receiving operating characteristic curve (AUROC) of 0.81 for 90-day suicide attempts; the other model tested had an AUROC of 0.68 for the same outcome. These AUROCs compared with an AUROC of 0.66 for a positive augmented screening indicator result.

**Meaning:**

Some existing suicide risk machine learning models are relevant and add value beyond existing screening practices in this population.

## Teaser

This prognostic study examines how well existing suicide risk models perform in a majority American Indian population.

## Introduction

American Indian and Alaska Native communities have shown remarkable strength and resilience despite the impact of colonization and assimilation. They have preserved and revitalized their languages and traditions and, as sovereign nations, have advocated for their rights and well-being. These communities have also been at the forefront of developing innovative solutions to health challenges, including suicide prevention.^1^

The rates of suicide in American Indian and Alaska Native populations consistently exceed those of other racial and ethnic groups in the US.^2^ These disparities have worsened recently.^3^ Suicide rates in American Indian and Alaska Native populations can be attributed to the confluence of historical, cultural, social, and economic factors perpetuating intergenerational trauma and disparities in accessing mental health resources.^4^ The devastating consequences of suicide on individuals, families, and communities underscore the urgent need for effective prevention strategies tailored to the unique needs and context of American Indian and Alaska Native populations.

Recent advances in machine learning offer to enhance our abilities to identify individuals at risk of suicide.^5,6,7,8^ Despite progress in the broader field of suicide risk identification, few existing models, including no screening or assessment tools, have been developed for or validated in American Indian and Alaska Native populations.^5,9^ This oversight has hindered the generalizability of these approaches and limited their potential impact on communities that arguably need them the most. Addressing this gap is critical to ensuring that promising approaches reach the populations most in need.

Applying models developed in one context to another presents potential pitfalls. Dataset shift occurs when the target population differs systematically from the population used to develop a model or tool, which can reduce accuracy and, in some cases, lead to harm.^10,11,12^ In suicide prevention, population differences in risk factors and protective factors may lead to models that overestimate or underestimate the risk of suicide.^10^ For example, in American Indian and Alaska Native populations, the burden of suicide is more concentrated in younger people compared with the general US population.^3^ Because age is often a key feature in existing models, this distributional shift may influence model performance.

Failure to recognize and address dataset shift can inadvertently exacerbate health disparities.^12,13,14^ Models that do not account for dataset shifts may mischaracterize the associations between risk factors and outcomes, leading to risk misclassification. Misclassification may, in turn, result in the misallocation of limited resources, thereby reinforcing existing disparities. For example, Obermeyer et al^15^ found that a model used to allocate resources underestimated needs in Black patients. Moreover, using models not sensitive to racial and ethnic differences may erode trust in health care systems, further inhibiting the uptake of services by individuals in need.

To avoid misclassification from dataset shift, we must assess the fit and performance of existing models and tools within new populations, particularly populations experiencing significant health inequities. This external validation is a critical step in model implementation, ensuring that prediction models hold up in different populations and under different conditions.^16^ The need for external evaluations has been demonstrated by several studies that identified failures by models to generalize among health systems,^17,18,19^ sexes,^19,20^ and academic vs community settings.^21^

Given the need to validate suicide risk models and tools in the populations where they will be used and the limited existing work doing this, we aimed to test the fit of 2 existing machine learning–based models for suicide risk in an American Indian population. We selected the models based on their validity in general populations and the availability of their coding methods. We examined whether existing, published suicide risk models are accurate and how well they perform when applied in a novel and majority American Indian population. We hypothesized that the models would not maintain their performance when tested in this way. By comparing model performance, we aimed to help develop more effective and culturally relevant risk identification tools for health systems that serve American Indian and Alaska Native populations. Identifying individuals at risk in this context may allow for more efficient allocation of already scarce resources and/or additional proactive outreach to prevent more serious and harmful outcomes.

## Methods

This prognostic study performed a secondary analysis of health care data from a single Indian Health Service (IHS) unit in the Southwestern US. The study built on a more than 40-year relationship among the White Mountain Apache Tribe, the local IHS unit, and the Center for Indigenous Health at the Johns Hopkins Bloomberg School of Public Health. The project was supported by a tribal resolution. The study was approved by the Johns Hopkins Bloomberg School of Public Health Institutional Review Board and the Phoenix Area Office of IHS Institutional Review Board as secondary data analysis under a Health Insurance Portability and Accountability waiver; therefore, informed consent was not required. The White Mountain Apache Tribe approved the study through the Health Advisory Board and Tribal Council. Reporting followed the Transparent Reporting of a Multivariable Prediction Model for Individual Prognosis or Diagnosis (TRIPOD) reporting guideline.

### Study Cohort

The cohort included patients 18 years or older with at least 1 encounter with the IHS unit between January 1, 2017, and December 31, 2021. All demographic characteristics, including race and ethnicity, were recorded in the electronic health record (EHR). The selection of a model focused on adults was based on local data indicating that increasingly American Indian and Alaska Native young adults are the most at-risk group, a trend that mirrors national patterns among American Indian and Alaska Native populations.^3^ All types of visits were used for feature construction (entire sample). A team of local physicians reviewed clinic visit types for relevant point-of-care suicide risk identification. Decisions were based on what physicians know about the type of visit and the types of interactions with patients during the visit. If a visit type held no relevance for suicide risk identification (eg, COVID-19–related vaccine or test visits), it was removed from the dataset for model performance evaluation (analytic sample).

### Outcomes

Our 2 primary outcomes were suicide attempts or suicide deaths. We used the definition of a suicide attempt from Simon et al^22^ but modified it to account for visits that had missing initial encounter codes. For these instances, we included cases in which the patient had an *International Statistical Classification of Diseases and Related Health Problems, Tenth Revision (ICD-10)* code for a subsequent encounter related to suicide attempts in the prior 90 days. We obtained suicide death data from records maintained by the tribe’s surveillance system.^23^ We examined outcomes at 30-, 60-, and 90-day intervals. The data was right-censored on October 3, 2021, to allow for a 90-day postvisit interval. We observed outcomes through December 31, 2021.

### Model Selection

We selected existing suicide risk models from the literature based on their applicability to adult populations. The first model was developed by Walsh and colleagues^6^ from Vanderbilt University (VU), with demonstrated accuracy in adult patients in inpatient setting, emergency departments (EDs), and ambulatory surgery settings. The other models were from the Mental Health Research Network (MHRN) and included models for general outpatient and specialty mental health care settings.^9,22,24^ Our primary results focus on the outpatient models from the MHRN, given that our analysis focused on a more general population given the lack of specialty mental health care in this setting. We obtained code to construct the model features from published articles, a publicly available GitHub repository, or the original research teams.

### Data Aggregation and Preprocessing

Data included EHR information for all patient interactions, including inpatient, outpatient, and ED visits, and on-site pharmacy prescription data. The data covered patient demographic characteristics, socioeconomic status, health insurance information at the time of the visit, diagnoses as indicated by *ICD-10* codes, prescribed medications, prescriptions filled at the on-site pharmacy, results from any ordered examinations or tests, and *Current Procedural Terminology* codes for all patient interactions with the health system. Additional details about data cleaning and accounting for missingness can be found in eAppendix 1 in Supplement 1.

### Tested Models

The VU model^6,25^ was trained using a random forest algorithm. Features in the model included demographic information, body mass index, 2022 Hierarchical Condition Category codes, *Current Procedural Terminology* codes, and Anatomical Therapeutic Chemical codes for medications. All codes were provided by the VU team. The number of potential features considered in the VU model was 828, of which 713 were included in their final model with variable importance scores greater than 0. We used all historical data and current visit data at the time of each encounter to create cumulative counts for these features in this dataset. Vanderbilt University trained the model using nonfatal suicide attempt as the outcome and validated it on both suicide attempt (primary outcome) and suicidal ideation (secondary outcome).

We focused on 2 MHRN models that leveraged logistic regression with penalized LASSO (least absolute shrinkage and selection operator) variable selection: a primary care model with an outcome of a nonfatal suicide attempt and a primary care model with an outcome of death by suicide. Model features included demographic characteristics, mental health and substance use diagnoses, past suicide attempts and other past injury or poisoning diagnoses, medications for mental health conditions, past inpatient and ED mental health care use, other medical diagnoses, and 9-item Patient Health Questionnaire (PHQ-9) scores (the model uses both individual scores from item 9, which range from 0-3, and from the sum of the first 8 items, which range from 0-24; higher scores represent greater symptom severity). The MHRN models started with 149 potential features and 164 interactions, and the final models after LASSO variable selection included between 30 and 104 features. We report the results of the primary care models for suicide attempts and deaths, and full results are available in eAppendix 2 in Supplement 1.

### Comparison With Existing Screening

We compared our evaluation metrics with existing screening practices. Universal screening in the ED was piloted in 2019 with men and fully implemented in January 2020. As part of these processes, all patients who visit the ED complete a screening measure. If their screening results are positive, they are further evaluated with a more in-depth risk assessment conducted by local behavioral health services. Before this and in other non-ED settings, the PHQ-9 is administered to assess risk. We considered a patient’s visit to have been identified by screening if it met any of the following criteria: (1) positive screen result (both acute and nonacute positive results on the ASQ) on the PHQ-9 or Ask Suicide-Screening Questions (ASQ)^26^ in the 90 days before the visit (the ASQ classifies risks as negative, nonacute positive, and acute positive; nonacute positive and acute positive both indicate suicide risk); (2) diagnosis of suicidal ideation in the 90 days before the visit; and (3) a recorded suicide attempt at any prior visit. The second and third criteria were included to avoid penalizing screening in cases in which suicide risk would have been sufficiently apparent, such that screening may not have been considered necessary.

### Statistical Analysis

For each model, we generated a predicted probability of subsequent suicide attempt and/or death for each health system contact in our data. We assessed each model’s discrimination by calculating the area under the receiver operating characteristic curve (AUROC). We also examined precision recall plots and calculated the positive predictive values (PPVs) and number needed to evaluate (NNE; or the total number of cases the model identifies to find a true case). We examined these metrics over a range of sensitivity values and generated risk concentration plots. To examine calibration, we created calibration plots and examined Spiegelhalter *z* statistics. A 2-sided *P* < .05 indicates the model is poorly calibrated.^27^ For all evaluation metrics (except risk concentration and calibration plots), we constructed 1000 samples using block bootstrapping, with patient-level replacement, to build CIs around these metrics. Then 95% CIs were calculated using the 2.5th and 97.5th percentiles across the estimates generated over the bootstrapped samples.

To compare why models performed differently, we assessed permutation feature importance in the IHS sample by measuring the mean decrease in AUROC for 90-day suicide attempt when the values of each feature are randomly shuffled (full details available in eAppendix 1 in Supplement 1). A large change in AUROC suggests that the feature contributed significantly to accurately predicting the probability of 90-day suicide attempt in the IHS sample. We then ranked each feature by the permutation feature importance value and compared that ranking with the ranking of feature importance based on the original model development samples. For the original model development sample for the VU model, we ranked variables by importance based on the decrease in node impurity, measured by the Gini index (for each variable, measures the amount that node impurity decreases in the child nodes across all nodes where the variable is used to make a split; variable importance scores; scores in the VU modeling study ranged from 0.0-49.8, with higher scores indicating greater importance, as reported in the original modeling study).^25^ For the MHRN models, we ranked variables based on the size of their β coefficients. All analyses were conducted in R, version 4.3.1 (R Foundation for Statistical Computing). Data were analyzed between October 6, 2022, and July 29, 2024.

### Sensitivity Analyses

We performed several sensitivity analyses, including varying methods of feature construction and stratifying results by visit year and patient sex. These analyses are included in eAppendix 3 in Supplement 1.

## Results

The entire study sample included 16 835 patients (mean [SD] age, 40.0 [17.5] years; 8660 [51.4%] female and 8175 [48.6%] male; 14 251 [84.7%] American Indian and 2584 [15.3%] of other races, including Asian, Black, White, or race recorded as other or unknown in the EHR). Overall, 324 patients (1.9%) had at least 1 suicide attempt (417 total attempts), with 37 deaths (0.2%). Full demographic characteristics of the individuals included in the entire dataset can be found in Table 1. After limiting the dataset to visits when potential suicide risk could be addressed (ie, removing vaccine appointments and record or notes updates), there were 331 588 included visits among 13 761 patients (81.7%), of whom 12 938 (94.0%) were identified by the EHR as American Indian or Alaska Native. In comparison, the validation sample for the VU model did not report any American Indian or Alaska Native patients (2080 of 77 973 patients [2.7%] are reported as other race) and reported 85 of 77 973 patients (0.1%) with an attempt included in the analysis. Similarly, among the 3 387 741 visits in the validation sample for the MHRN primary care model, there were 37 717 visits (1.1%) in which the patient was American Indian or Alaska Native, 8688 visits (0.3%) in which there was a suicide attempt in the 90 days following the visit, 455 visits (<0.1%) in which there was a suicide death in the 90 days following the visit, and 2 839 199 visits (83.8%) by a patient with private insurance. Other demographic characteristics reported in Table 1 were not directly comparable with the results reported from the VU or MHRN validation samples.

**Table 1.** Characteristics for the 16 835 Patients Included in the Dataset^a^

Characteristic	No. (%) of patients^b^		
	All (N = 16 835)	No attempt or death (n = 16 482)	Attempt or death (n = 353)
Demographics			
Age at first visit, mean (SD), y	40.2 (17.2)	40.5 (17.2)	30.0 (11.0)
Sex			
Female	8660 (51.4)	8493 (51.5)	167 (47.3)
Male	8175 (48.6)	7989 (48.5)	186 (52.7)
Race			
American Indian or Alaska Native^c^	14 251 (84.7)	13 899 (84.3)	352 (99.7)
Other^d^	2584 (15.3)	2583 (15.7)	1 (0.3)
Outcomes			
Attempted suicide	324 (1.9)	NA	324 (91.8)
Death by suicide	37 (0.2)	NA	37 (10.5)
Existing screening			
Positive suicide screen	785 (4.7)	648 (3.9)	137 (38.8)
Positive depression screen	1697 (10.1)	1497 (9.1)	200 (56.7)
Insurance			
Not enrolled	4868 (28.9)	4851 (29.4)	17 (4.8)
Medicaid	9398 (55.8)	9067 (55.0)	331 (93.8)
Private	1858 (11.0)	1848 (11.2)	10 (2.8)
Medicare	2297 (13.6)	2281 (13.8)	16 (4.5)

Abbreviation: NA, not applicable.

^a^ Numbers of 10 or less (and associated *P* values) are suppressed to protect patient privacy.

^b^ Unless otherwise indicated.

^c^ This is the percentage in the full dataset, but many of the visits by patients of races other than American Indian or Alaska Native were to clinics excluded from our analytic sample. The analytic sample in which the models were evaluated was 94.0% American Indian or Alaska Native.

^d^ Other races were Asian, Black, White, and race recorded as other or unknown in the electronic health record.

Among the 388 suicide attempts that occurred with at least 90 days of preattempt observation time, 280 attempts (72.2%) were preceded by an IHS contact at which a point-of-care suicide intervention was deemed feasible (eg, ED or clinic visit vs a vaccine appointment). However, only 124 attempts (32.0%) were identified based on the modified existing screening approach. This number ranged from 9 of 62 attempts (14.5%) in 2017 to 32 of 72 attempts (44.4%) in 2020. Of the attempts that had contact with IHS in the prior 90 days, 233 of 280 (83.2%) had visits to the ED, and 200 of 280 (71.4%) had other IHS system contacts. Among the 36 suicide deaths that occurred with at least 90 days of predeath observation time, 18 deaths (50.0%) were preceded by an IHS contact at which a point-of-care suicide intervention was deemed feasible.

Model performance across all models and outcomes evaluated can be found in Table 2. Using our existing screening definition, we estimated that the AUROC values were 0.66 (95% CI, 0.63-0.70) for 90-day suicide attempts and 0.48 for 90-day suicide death (bootstrapped 95% CIs were unable to be calculated because none of the suicide deaths in our sample were identified by current screening). The VU model showed lower discrimination than the MHRN primary care model, with an AUROC value of 0.68 (95% CI, 0.64-0.72) compared with an AUROC value of 0.81 (95% CI, 0.77-0.85) for 90-day suicide attempts for the MHRN model. Performance for all models differed significantly by visit year, with estimated performance improving each year for the existing screening and the MHRN model and having the highest performance in 2019 for the VU model (eTable 4 in Supplement 1). For 90-day death by suicide, the VU model had an AUROC value of 0.63 (95% CI, 0.46-0.80) compared with 0.86 (95% CI, 0.78-0.92) for the MHRN model. The existing screening definition did not identify any suicide deaths, even in the most extended time window (sensitivity was 0.0%). Results were consistent across 30-, 60-, and 90-day time windows for both models and both suicide attempts and deaths. The ROC curves are displayed in the Figure.

**Table 2.** Model Performance Comparing Existing Suicide Risk Models With Existing Screening at an Indian Health Service Facility in the Southwest for Suicide Attempts and Suicide Deaths for 331 558 Patient Visits

Variable	VU	MHRN primary care	Existing screening^a^
**AUROC (95% CI)**			
Time window of suicide attempt, d			
30	0.69 (0.65-0.74)	0.84 (0.80-0.88)	0.71 (0.66-0.75)
60	0.68 (0.64-0.72)	0.82 (0.78-0.86)	0.67 (0.64-0.71)
90	0.68 (0.64-0.72)	0.81 (0.77-0.85)	0.66 (0.63-0.70)
Time window of suicide death, d			
30	0.82 (0.66-0.93)	0.86 (0.78-0.95)	0.48^b^
60	0.67 (0.48-0.83)	0.87 (0.79-0.94)	0.48^b^
90	0.63 (0.46-0.80)	0.86 (0.78-0.92)	0.48^b^
**No. needed to evaluate 90-d suicide attempt (95% CI)**			
Selected sensitivity, %			
35	172 (122-232)	50 (28-74)	39 (30-52)
50	177 (132-234)	61 (44-101)	NA
70	216 (165-278)	123 (84-168)	NA
90	253 (205-321)	189 (144-264)	NA
**No. needed to evaluate 90-d suicide death (95% CI)**			
Selected sensitivity, %			
33	4439 (199-11979)	1454 (133-4287)	NA
50	3663 (309-10849)	1440 (146-3904)	NA
70	5312 (2116-10829)	1622 (756-4229)	NA
90	5601 (2955-10961)	2088 (982-6250)	NA

Abbreviations: AUROC, area under the receiver operating characteristic curve; MHRN, Mental Health Research Network; NA, not applicable; VU, Vanderbilt University.

^a^ For the current screening scenario, the number needed to evaluate could not be assessed for suicide attempt except at the baseline sensitivity (because there is no alternative cut point to increase the sensitivity) and was not able to be calculated for suicide death because no suicide deaths were identified from current screening.

^b^ Bootstrapped CIs for the current screening AUROC for the suicide death outcome were unable to be calculated because none of the suicide deaths in our sample were identified by current screening. Thus, when taking bootstrapped CIs, the AUROC remained fixed.

**Figure.**
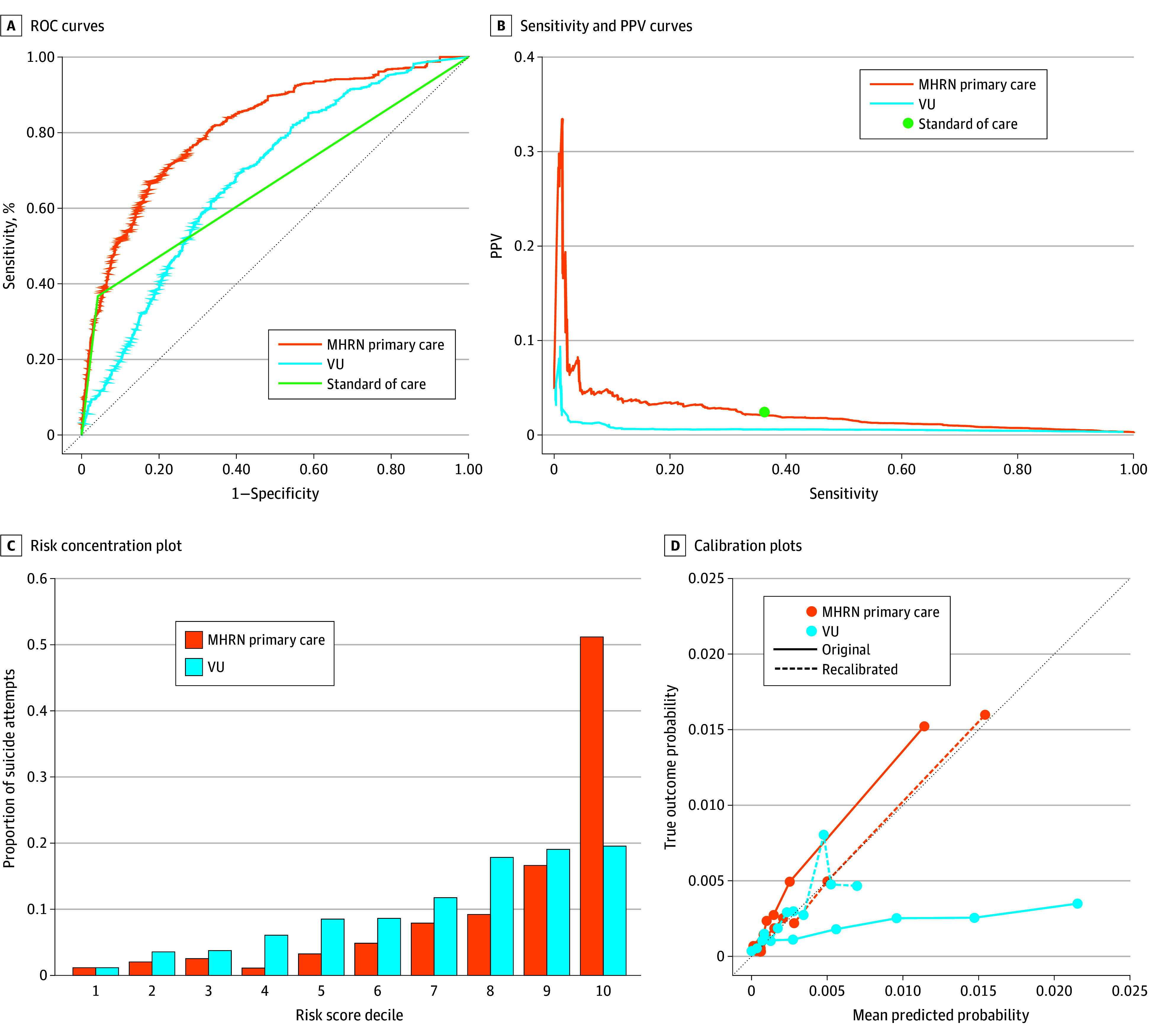
Receiver Operating Characteristic (ROC) Curves for Mental Health Research Network (MHRN) Primary Care, Vanderbilt University (VU), and Existing Screen for 90-Day Suicide Attempts for 331 558 Patient Visits PPV indicates positive predictive value.

For the outcome of 90-day suicide attempt, the existing screening definition had a sensitivity of 36.7% (95% CI, 29.8%-44.3%), a PPV of 0.025 (95% CI, 0.019-0.033), and an NNE of 39 (95% CI, 30-52). Setting a threshold value for both the VU and the MHRN models to achieve an equal sensitivity resulted in a PPV and NNE of 0.020 (95% CI, 0.013-0.036) and 50 (95% CI, 28-74) for the MHRN primary care model and 0.006 (95% CI, 0.004-0.008) and 172 (95% CI, 122-232) for the VU model. The PPV remained relatively stable for the MHRN models, as sensitivity increased past 36.7% (Figure). Risk concentration plots for 90-day suicide attempts are shown in the Figure. For the VU model, 193 visits followed by a suicide attempt within 90 days (19.6%) were in the top decile of predicted probabilities. For the MHRN primary care model, 505 visits followed by a suicide attempt within 90 days (51.2%) were in the top decile of predicted probabilities.

Calibration curves for 90-day suicide attempts are shown in the Figure. For the outcome of 90-day suicide attempts, the Spiegelhalter *z* score was −89.9 (*P* < .001) for the VU model and 15.5 (*P* < .001) for the MHRN primary care model. After recalibration using isotonic regression, the cross-validated Spiegelhalter *z* score was 0.18 (*P* = .86) for the VU and 0.19 (*P* = .85) for the MHRN primary care model.

Finally, we examined differences in AUROC values for 90-day suicide attempts using our permutation feature importance procedures (Table 3). Among the top 10 most important features in the original analysis for the VU model, only 4 remained in the top 10 for the IHS sample: major depressive, bipolar, and paranoid disorder diagnoses; ED visits, moderate level of medical decision-making; outpatient medical visits (30-39 minutes); and drug and alcohol use disorder diagnoses. For the MHRN primary care model, 5 of the original top 10 most important features remained in the top 10 in the IHS sample: depressive disorder diagnosis in the previous 5 years; any previous suicide attempt in the previous 5 years; drug use disorder diagnosis in the previous 5 years; drug use disorder diagnosis in the previous 5 years, not including the current visit; and alcohol use disorder diagnosis in the previous 5 years. Additional displays of these rankings, including the top features in the IHS sample and their rankings in the MHRN and VU models, are included in eTable 1 in Supplement 1.

**Table 3.** Comparison of Top 10 Features and Their Importance Across Samples for 90-Day Suicide Attempt

Variable	Model development sample		IHS sample	
	Rank	Value^a^	Rank	AUROC change^b^
**VU model feature importance**				
Age	1	49.8	12	0.0021
Major depressive, bipolar, and paranoid disorders	2	49.4	1	0.0733
Body mass index	3	47.6	810	−0.0005
Emergency visit of moderate complexity	4	20.2	5	0.0061
Area Deprivation Index^c^	5	18.9	46	0.0001
Emergency visit of high complexity	6	16.5	17	0.0014
Selective serotonin reuptake inhibitor prescriptions	7	15.5	825	−0.0015
Emergency visit of very high complexity	8	14.4	15	0.0015
Outpatient visit lasting 30-39 min	9	13.5	8	0.0033
Drug or alcohol use dependence disorders	10	13.2	3	0.0176
**MHRN primary care model feature importance**				
Depression diagnosis in previous 5 y^d^	1	1.72	1	0.0779
History of suicide attempt in previous 5 y^d^	2	1.67	6	0.0169
Drug use disorder diagnosis in previous 5 y^d^	3	1.30	3	0.0377
Interaction of history of suicide attempt in previous 5 y and depression diagnosis in previous 5 y^d^	4	−1.00	22	0.0008
Drug use disorder diagnosis, previous 5 y^e^	5	−0.81	10	0.0063
Interaction between ages 13-17 y and female sex	6	0.71	53	0.0000
Alcohol use disorder diagnosis in previous 5 y	7	0.58	5	0.0184
Interaction of report of “not at all” for question 9 (suicidal ideation) on PHQ-9 at index visit and depression diagnosis in previous 5 y^d^	8	−0.57	49	0.0000
Report of “nearly every day” for question 9 (suicidal ideation) on PHQ-9 in previous 1 y	9	0.49	46	0.0000
Interaction of history of suicide attempt in previous 5 y and drug dependence diagnosis in previous 5 y^d^	10	−0.49	88	−0.0002

Abbreviations: AUROC, area under the receiver operating characteristic curve; IHS, Indian Health Service; MHRN, Mental Health Research Network; PHQ-9, 9-item Patient Health Questionnaire; VU, Vanderbilt University.

^a^ Feature importance in the VU model development samples is measured using the Gini index, and feature importance in the MHRN model is defined as the absolute value of the β coefficient. These values are not directly comparable with each other or with the decrease in AUROC reported in this study.

^b^ AUROC change is the mean decrease in AUROC value when that variable is randomly shuffled while leaving other variables unchanged across 25 iterations. Large values suggest the variable was important to accurately predict the probability of 90-day suicide attempt in the IHS sample.

^c^ Area Deprivation Index is a continuous score measuring neighborhood-level socioeconomic disadvantage. Higher scores indicate increased disadvantage. Details on the score used in the VU model are in the original model publication.^25^

^d^ MHRN model variable includes index visit.

^e^ MHRN model variable does not include index visit.

## Discussion

This study aimed to evaluate the performance of existing suicide risk models on routinely collected EHR data on a different population. After recalibration, the models developed by the MHRN accurately detected and stratified the risk of suicide attempts and deaths among a population of American Indian patients across visit settings. Our results provide the IHS, a health system that serves more than 2.6 million people, an important tool that may contribute to mitigating suicide-related health inequities.

This study builds on key findings from previous work with American Indian and Alaska Native communities. First, the demonstrated performance of the MHRN models is consistent with recent findings showing this model’s applicability in an American Indian and Alaska Native population in Alaska.^9^ Shaw and colleagues^9^ showed that the MHRN models, when applied to an EHR, were highly accurate. Our work extends the generalizability of these models, including generalization to the Resource and Patient Management System (RPMS), a different EHR but one that is used across all IHS facilities. Although the RPMS is listed as a “critical federal legacy system in need of modernization,”^28^ it currently serves more than 2.2 million patients. Demonstrating the utility of the MHRN models in this population and with RPMS data can potentially advance patient services in the near term. This work also advances previous studies by our group building and implementing suicide risk models in partnership with American Indian and Alaska Native communities.^5,29,30^

Although the generalization of the MHRN models is notable, all models were poorly calibrated initially, demonstrating the importance of recalibration when applying models to new populations. The MHRN models also relied on constructed features often based on data that some health systems may find challenging to obtain (eg, the reliance on the PHQ-9 item level responses, which IHS did not have in the current analysis). Reliance on feature construction in this way may prove challenging for some health systems to implement. The relatively poor performance of the VU model is also interesting. We hypothesize that the method of feature construction influenced this performance difference. The VU model relied more on counts of administrative and claims data. Such features, although potentially easier to obtain, may be subject to variability in practitioner and health system practice, which can affect model performance.^31^ The MHRN models included fewer features, chosen based on theory and known suicide risk and protective factors. Although some in the machine learning world advocate for a “kitchen sink” approach similar to the VU model, some research suggests that this approach may fall short, particularly when models are trained to predict a proxy for the true outcome (eg, diagnosis codes are an imperfect proxy for true suicide attempts).^32^

We chose to compare model performance with existing screening practices, including diagnoses of recent suicidal ideation, past suicide attempts, and positive depression and suicide screening data. We assume that clinicians use these tools, as well as additional information, to inform their judgment of a patient’s potential risk of suicide. In 2020, the hospital in this study adopted universal suicide risk screening using the ASQ^26^ for any patient seen in the ED. Our results show that the machine learning models used in this study had substantially higher AUROC values than existing approaches, even when compared with a substantial uptick in ASQ administration (eTable 5 in Supplement 1).

In previous studies,^33,34^ the ASQ has shown high concordance with other measures and accurately identifies future suicide-related behaviors in pediatric ED patients, those presenting with psychiatric problems, and those with medical emergencies. However, the ASQ has never been validated in American Indian and Alaska Native populations, and, as with any intervention, even a brief screening tool, there can be implementation challenges. These challenges may include when and how the tool is administered, how the questions are asked, and even if they are asked at all. In the context of imperfect tools to identify suicide risk, determining how to use the tools we have—together—is vital to advancing suicide prevention efforts. Future research should validate the ASQ independently and compare its performance with other markers of suicide risk.

Despite racial and ethnic bias in many algorithms used to support clinical decision-making,^15^ including suicide,^13^ few models are externally validated on new populations. This lack of external validation is true of tools recommended by hospital regulatory bodies. For example, the Joint Commission recommended that patients with behavioral issues be evaluated with “validated” screening tools,^35^ yet no validation studies had been performed in American Indian and Alaska Native populations. Use of unvalidated tools can exacerbate existing health inequities and leave populations to use tools that are not designed to meet their specific needs.

Recently, authors have pointed to key strategies to improve the effect of machine learning models on health equity.^36^ Some key strategies identified include improving the diversity, quality, or quantity of data and engaging communities at every stage of model development and translational phases.^37^ Our work represents a collaboration among university, tribal, and federal partners. The results presented in this article represent a step toward building a tool designed by and for tribal communities. Testing the applicability of models in new populations helps to expand our understanding of how best to use these tools to advance health equity.

### Limitations

This study has some limitations. Relying on EHR data alone can be problematic. Diagnosis codes and prescription records include errors and bias. Such bias is likely in the IHS context, just as it is in any EHR system. The impreciseness in our features and outcomes may have contributed to errors in model performance. We were also limited in the features used across health systems (eg, PHQ-9, item-level data). This limitation prevented strict comparisons. However, we approached this concern via sensitivity analyses that allowed us to compare model performance under different assumptions. We are further limited in our generalizability because we focused on an adult population in a single tribal context. Tribes and other American Indian and Alaska Native communities are diverse and heterogeneous. Our results may not generalize to other American Indian and Alaska Native settings. Additionally, death by suicide is a sufficiently rare outcome for which our estimates of model performance for this outcome had high statistical uncertainty, although our estimates were broadly consistent with results for suicide attempt, which had much less statistical uncertainty. Finally, although we worked within a relatively closed health care system, it is possible that our data sources to define suicide attempts and deaths by suicide were not complete; thus, it is impossible to know whether we undercounted these outcomes in our models.

## Conclusions

Existing risk identification models for suicide prevention hold promise when applied to new contexts, including American Indian and Alaska Native populations. The MHRN models tested in this prognostic study performed better than relying on suicide screening, reports of recent suicidal ideation, and history of attempts. The MHRN model was significantly more accurate than the VU model. These differences highlight the need for caution when applying risk models to new populations. Future work will compare the performance of the models tested here with a population-specific model, engaging multiple levels of stakeholders, before implementing these models in clinical care pathways.

## Notes

Supplement 1. **eAppendix 1.** Additional Methods Details **eTable 1.** Feature Importance Results for 90-Day Suicide Attempt **eAppendix 2.** Supplementary Results **eTable 2.** MHRN Specialty Mental Health Care Model Performance Compared to Existing Screening at an IHS Facility in the Southwest for Suicide Attempts and Suicide Deaths (N = 351,588) **eAppendix 3.** Sensitivity Analysis Results **eTable 3.** MHRN Model Performance Comparing Two Ways of Handling Missing PHQ-9 Data (N = 351,558) **eTable 4.** Vanderbilt Model Performance Comparing Predictions Made Including or Excluding Data From the Current Visit (N = 351,558) **eTable 5.** Model Performance Stratified by Year of Visit (N = 351,558) **eAppendix 4.** Comparison of Demographic Characteristics **eTable 6.** Model Performance Comparing Predictions for Males and Females (N = 351,558) **eTable 7.** Comparing Demographic Characteristics Across Patient Populations Used in Model Development and the Current Study

Supplement 2.
Data Sharing Statement
