# Supplementary material for: Performance of Machine Learning Suicide Risk Models in an American Indian Population
Source: JAMA Netw Open. 2024 Oct 14;7(10):e2439269. doi: 10.1001/jamanetworkopen.2024.39269 (PMC11474420; doi:10.1001/jamanetworkopen.2024.39269)
Supplement: Supplement 1. — eAppendix 1. Additional Methods Details eTable 1. Feature Importance Results for 90-Day Suicide Attempt eAppendix 2. Supplementary Results eTable 2. MHRN Specialty Mental Health Care Model Performance Compared to Existing Screening at an IHS Facility in the Southwest for Suicide Attempts and Suicide Deaths (N = 351,588) eAppendix 3. Sensitivity Analysis Results eTable 3. MHRN Model Performance Comparing Two Ways of Handling Missing PHQ-9 Data (N = 351,558) eTable 4. Vanderbilt Model Performance Comparing Predictions Made Including or Excluding Data From the Current Visit (N = 351,558) eTable 5. Model Performance Stratified by Year of Visit (N = 351,558) eAppendix 4. Comparison of Demographic Characteristics eTable 6. Model Performance Comparing Predictions for Males and Females (N = 351,558) eTable 7. Comparing Demographic Characteristics Across Patient Populations Used in Model Development and the Current Study [file jamanetwopen-e2439269-s001.pdf]

Supplementary Online Content

Haroz EE, Rebman P, Goklish N, et al. Performance of machine learning suicide risk models in American Indian and Alaska Native populations. *JAMA Netw Open*. 2024;7(10):e2439269. doi:10.1001/jamanetworkopen.2024.39269

eAppendix 1. Additional Methods Details

eTable 1. Feature Importance Results for 90-Day Suicide Attempt

eAppendix 2. Supplementary Results

eTable 2. MHRN Specialty Mental Health Care Model Performance Compared to Existing Screening at an IHS Facility in the Southwest for Suicide Attempts and Suicide Deaths (N=351,588)

eAppendix 3. Sensitivity Analysis Results

eTable 3. MHRN Model Performance Comparing Two Ways of Handling Missing PHQ-9 Data (N=351,558)

eTable 4. Vanderbilt Model Performance Comparing Predictions Made Including or Excluding Data From the Current Visit (N=351,558)

eTable 5. Model Performance Stratified by Year of Visit (N=351,558)

eAppendix 4. Comparison of Demographic Characteristics

eTable 6. Model Performance Comparing Predictions for Males and Females (N=351,558)

eTable 7. Comparing Demographic Characteristics Across Patient Populations Used in Model Development and the Current Study

This supplementary material has been provided by the authors to give readers additional information about their work.

© 2024 Haroz EE et al. *JAMA Network Open.*

eAppendix 1. Additional Methods Details

Additional information on data cleaning and pre-processing

Data cleaning was minimal to approximate the accuracy of the models in real-time implementation. The MHRN models used the 9th item on the Patient Health Questionnaire-9 (PHQ-9). Item 9 asks about suicide ideation in the last two weeks and was highly important in the original algorithms. However, item-level data for the PHQ-9 was unavailable in IHS data. For patients with a suicide attempt or death in the 90 days following the visit, we set the value for the 9th item as '0,' denoting no endorsement of suicidal ideation. Given the association between the variable and the outcome in the original algorithm, handling missing data in this way created a lower bound on the model's performance. As a sensitivity analysis, we also calculated model performance setting the 9th item to '1' but results remained consistent (see Supplemental Section S3 below). Missing data for other features in the MHRN models was handled by indicator variables denoting missing data for particular variables. Only the BMI feature from the VU model had missing data, which was 1.1% across observations. Single-mean imputation was used to impute missing BMI.

Additional methods related to how tested models were constructed

For the VU model, we built ATC features by mapping the medication's name, dosage, and administration route, stored as text, to National Drug Codes (NDC). We mapped NDCs to ATC codes using files provided by the Vanderbilt team. We constructed HCC features from ICD-10 codes using a file mapping ICD-10 codes to HCC features provided by Vanderbilt University. In the original model validation, HCC, CPT, and ATC features were based on cumulative counts

© 2024 Haroz EE et al. *JAMA Network Open.*

from the previous 5 years of EHR data for each patient. As our dataset spanned a total of five years, all previous data for each visit was used to construct cumulative counts. The original feature definitions for the VU model did not include data from the current visit in constructing the cumulative count features. However, we included current visit data for the VU model in feature construction to make performance more comparable with the MHRN models. This choice was examined in a sensitivity analysis included in Supplemental Section S3 below.

For the MHRN models, we built medication features by mapping the generic medication name to medication categories, using files provided by MHRN. Most features for diagnoses, medication use, healthcare utilization, and PHQ-9 scores using overlapping temporal categories (e.g., 90 days before the visit, 1 year before the visit, 5 years before the visit/any time) are defined as dichotomous indicator variables.

Comparison of feature importance across models

To assess feature importance across models, we utilized a permutation importance method, in which we randomly shuffled the observed values for one variable at a time, while leaving the other variables unchanged. We then calculated the resulting AUROC differences for each variable. This procedure was conducted 25 times and the mean change in AUROC across these iterations was calculated for each variable. We used 90-day suicide attempts as our outcome for calculating permutation importance. Larger shifts in AUROC values following random shuffling of a feature's values suggested the feature was more important in accurately predicting outcome probability.(28) The results of these analyses are displayed in Table 3 of the paper.

© 2024 Haroz EE et al. *JAMA Network Open.*

In addition, here we display the results of the permutation importance slightly differently. Supplemental Table 1, the top features in the IHS sample are listed with their corresponding rankings, values, and change in AUROC values in the VU and MHRN original validation samples.

eTable 1. Feature Importance Results for 90-Day Suicide Attempt

Top 10 Features in Original Model Development Sample						
	**VU Model Development Sample**			**MHRN Primary Care Model Development Sample**		
**Top 10 features in IHS sample**	**Rank**	**Value[a]**	**AUROC Change[b]**	**Rank**	**Value[a]**	**AUROC Change[b]**
1. Major Depressive, Bipolar, and Paranoid Disorders	2	49.36	0.0733	1	1.719	0.0779
2. Emergency Visit, Low Complexity	15	10.46	0.0194	86	-0.018	0.0411
3. Drug/Alcohol Use Disorders	10	13.16	0.0176	3	1.296	0.0377
4. Outpatient Visit, 20-29 Minutes	12	12.37	0.0133	65	-0.100	0.0359
5. Emergency Visit, Moderate Complexity	4	20.17	0.0061	7	0.580	0.0184
6. Aspiration and Specified Bacterial Pneumonias	35	5.94	0.0039	2	1.668	0.0169
7. Emergency Visit, Straightforward Complexity	70	3.72	0.0038	90	-0.012	0.0142
8. Outpatient Visit, 30-39 Minutes	9	13.53	0.0033	15	-0.405	0.0082
9. Diazepine, Oxazepine, Thiazepine, and Oxepine Prescriptions	14	10.55	0.0032	34	0.202	0.0071
10. Other Antipsychotic Prescriptions	22	8.29	0.0023	5	-8.15	0.0063

Notes:
a. Feature importance in the VU model development samples is measured using the GINI index and feature importance in the MHRN model is defined as the absolute value of the beta coefficient. These values are not directly comparable to each other or to the decrease in AUROC reported in our study.
b. AUROC Change is the mean decrease in AUROC value when that variable is randomly shuffled while leaving other variables unchanged across 25 iterations. Large values suggest the variable was important to accurately predict the probability of 90-day suicide attempt in the IHS sample.
c. MHRN Model variable includes index visit
d. MHRN Model variable does not include index visit

Description of Sensitivity Analyses

We examined differences in model performance by varying how we constructed the PHQ-9 features for the MHRN models. Additionally, we examined differences in the performance of the VU model when including vs excluding the current visit data in generating predictions. We additionally examined results stratified by year of visit and patient's sex.

eAppendix 2. Supplementary Results

MHRN Specialty Mental Health Care Model Results

The MHRN research team trained four models (24) using logistic regression with penalized LASSO (least absolute shrinkage and selection operator) variable selection. The MHRN models included two model developed for primary care, and two model developed for specialty mental health visits, with one model from each setting predicting suicide attempts and one predicting suicide death. We reported on the models for primary care settings in our main results. Here we report on outcomes from the models for the specialty mental health care setting.

The MHRN specialty mental health care models had AUROC values of 0.81 [95% CI: 0.78, 0.85] for 90-day suicide attempt and a AUROC value of 0.78 [0.43, 0.90] for 90-day death by suicide. The AUROC value for 90-day suicide attempt was significantly better than our comparison to existing screening practices.

Based on our permutation importance procedures for the MHRN specialty mental health care model, only three of the top ten most important features in the original model remained in the top ten most important features in the new dataset. These features included: Depressive disorder diagnosis at any point in the previous 5 years; Drug use disorder diagnosis at any point in the previous 5 years; and Alcohol use disorder diagnosis at any point in the previous 5 years.

© 2024 Haroz EE et al. *JAMA Network Open.*

eTable 2. MHRN Specialty Mental Health Care Model performance compared to existing screening at an IHS facility in the Southwest for suicide attempts and suicide deaths (N=351,588)

**AUROC [95% CI]**		
	MHRN Specialty Mental Health	Existing Screening
30-Day Suicide Attempt	0.84 [0.79, 0.87]	0.71 [0.66, 0.75]
60-Day Suicide Attempt	0.82 [0.78, 0.85]	0.67 [0.64, 0.71]
90-Day Suicide Attempt	0.81 [0.78, 0.85]	0.66 [0.63, 0.70]
30-Day Suicide Death	0.75 [0.37, 0.94]	0.48*
60-Day Suicide Death	0.78 [0.42, 0.92]	0.48*
90-Day Suicide Death	0.78 [0.43, 0.90]	0.48*

Number Needed to Evaluate: 90-Day Suicide Attempt [95% CI]

Selected Sensitivity	MHRN Specialty Mental Health	Existing Screening[a]
35%	49 [32, 80]	39 [30, 52]
50%	69 [46, 96]	-
70%	124 [88, 173]	-
90%	184 [145, 259]	-

Number Needed to Evaluate: 90-Day Suicide Death [95% CI]

Selected Sensitivity	MHRN Specialty Mental Health	Existing Screening[a]
33%	1479 [65, 7989]	-
50%	1783 [150, 5794]	-
70%	2804 [910, 8271]	-
90%	4286 [1204, 8734]	-

© 2024 Haroz EE et al. *JAMA Network Open.*

* Bootstrapped confidence intervals for the current screening AUROC for the suicide death outcome were unable to be calculated as none of the suicide deaths in our sample were identified by current screening. Thus, when taking bootstrapped confidence intervals, the AUROC remained fixed.

[a] Blank values indicate the measure was not applicable. For the current screening scenario, NNE could not be evaluated for suicide attempt except at the baseline sensitivity (as there is no alternative cut-point to increase the sensitivity) and was not able to be calculated for suicide death, as no suicide deaths were identified from current screening.

© 2024 Haroz EE et al. *JAMA Network Open.*

eAppendix 3. Sensitivity Analysis Results

Varying Construction of Missing PHQ-9 Features in the MHRN Models

In this sensitivity analysis, we varied the construction of the features for the 9th item, by setting the missing PHQ-9 9th item response to '1,' indicating several days of suicide ideation for individuals who had a positive label. This is equivalent to assuming patients who had a subsequent attempt or death would have reported current suicidal ideation on the PHQ-9 and no other patients would have reported current suicidal ideation. This assumption likely overestimates true model performance but serves as an upper bound for how these models would perform in this setting with access to data on the 9th item.

Overall, results were similar regardless of how we constructed the features corresponding to the 9th item of the PHQ-9 (Supplemental Table 2). For 90-day suicide attempt in the primary care models, our main analysis had an AUROC of 0.81 [0.77, 0.85], while our sensitivity analysis had an AUROC of 0.81 [0.77, 0.85]. For 90-day suicide death, the main analysis had an AUROC of 0.86 [0.78, 0.92], while our sensitivity analysis had an AUROC of 0.86 [0.78, 0.92].

© 2024 Haroz EE et al. *JAMA Network Open.*

eTable 3. MHRN model performance comparing two ways of handling missing PHQ-9 data. (N=351,558)

**AUROC [95% CI]**				
	MH Specialty Care – Upper Bound	MH Specialty Care – Lower Bound	Primary Care– Upper Bound	Primary Care– Lower Bound
30-Day Suicide Attempt	0.84 [0.80, 0.88]	0.84 [0.79, 0.87]	0.84 [0.80, 0.88]	0.84 [0.80, 0.88]
60-Day Suicide Attempt	0.82 [0.78, 0.86]	0.82 [0.78, 0.85]	0.82 [0.78, 0.86]	0.82 [0.78, 0.86]
90-Day Suicide Attempt	0.82 [0.78, 0.85]	0.81 [0.78, 0.85]	0.81 [0.77, 0.85]	0.81 [0.77, 0.85]
30-Day Suicide Death	0.75 [0.37, 0.94]	0.75 [0.37, 0.94]	0.86 [0.78, 0.95]	0.86 [0.78, 0.95]
60-Day Suicide Death	0.78 [0.42, 0.92]	0.78 [0.42, 0.92]	0.87 [0.79, 0.94]	0.87 [0.79, 0.94]
90-Day Suicide Death	0.78 [0.43, 0.90]	0.78 [0.43, 0.90]	0.86 [0.78, 0.92]	0.86 [0.78, 0.92]

Upper bound refers to the sensitivity analysis in which we assumed those with missing scores for the 9th item of the PHQ-9 data who subsequently attempted or died by suicide would have reported suicidal ideation at any visits in the previous 90 days. Lower bound refers to our main analysis, in which we assumed those with missing scores for the 9th item of the PHQ-9 would not have reported any suicidal ideation.

Inclusion of Current Visit Data in VU Model

The original validation of the VU model did not include data from the current visit in feature construction. This corresponds to a use case in which predicted probabilities are calculated and presented to clinicians at the initial point of an encounter with a patient. However, the original validation of the MHRN models utilized data from the current visit in feature construction. Inclusion of data from the current visit provides additional information and has the

© 2024 Haroz EE et al. *JAMA Network Open.*

potential to make the model more accurate. As a result, to make results between the two models more comparable, we utilized current visit data in the main results. Here we present results for VU based on feature construction without information from the current visit.

No significant differences for any outcome were observed when comparing predictions made with and without data from the index visit (Supplemental Table 3). AUC values for our main outcome of 90-day suicide attempt were 0.68 [0.64, 0.72] for our main analysis, including the current visit, and 0.68 [0.64, 0.73] for our sensitivity analysis, excluding the current visit.

© 2024 Haroz EE et al. *JAMA Network Open.*

eTable 4. Vanderbilt Model performance comparing predictions made including or excluding data from the current visit. (N=351,558)

**AUROC [95% CI]**		
	Including Current Visit	Excluding Current Visit
30-Day Suicide Attempt	0.69 [0.65, 0.74]	0.68 [0.64, 0.73]
60-Day Suicide Attempt	0.68 [0.64, 0.72]	0.67 [0.63, 0.71]
90-Day Suicide Attempt	0.68 [0.64, 0.72]	0.68 [0.64, 0.72]
30-Day Suicide Death	0.82 [0.66, 0.93]	0.83 [0.66, 0.94]
60-Day Suicide Death	0.67 [0.48, 0.83]	0.67 [0.47, 0.83]
90-Day Suicide Death	0.63 [0.46, 0.80]	0.63 [0.45, 0.80]

Model Performance by Year

Across the years of data included in our analysis, three major factors could have contributed to differences in model performance by year. First, our analysis includes data both before and after the start of the COVID-19 global pandemic. COVID-19 may have resulted in changes to the underlying distributions of features or associations between features and labels and thus, changes in model performance. Additionally, during our observation time period, significant changes in screening practices occurred within our healthcare setting, such that the number of recorded screenings in the data increased significantly. Finally, as our data was limited to data spanning five years, we are limited in our ability to match the feature construction procedures for the VU model, in which they used five years of previous data for all patients, and some MHRN features. We would expect to see significant increases in performance over time if this difference in data availability explains the comparatively poorer performance in our dataset

© 2024 Haroz EE et al. *JAMA Network Open.*

compared to the original validation data. Here we present results of model performance for suicide attempts, stratified by year from 2017 to 2021. We considered the total number of deaths by suicide per year (ranging from 2-6 per year) to be insufficient to accurately assess outcomes on death outcomes stratified by year.

There were significant differences in discrimination for our existing screening measure and both the VU and MHRN Primary Care model. The estimated AUROC for existing screening for 90-day suicide attempt increased from 0.56 [0.54, 0.58] in 2017 to 0.74 [0.70, 0.78] in 2020. The VU model had the highest AUROC value in 2019, with a value of 0.75 [0.72, 0.77] for 90-day suicide attempt. However, discrimination was similar in the first and final years of the dataset, with AUROC values of 0.65 [0.61, 0.68] and 0.68 [0.63, 0.72], respectively. Finally, the estimated AUROC for the MHRN Primary Care model increased each year, from 0.77 [0.74, 0.80] in 2017 to 0.88 [0.85, 0.90] in 2021.

© 2024 Haroz EE et al. *JAMA Network Open.*

eTable 5. Model performance stratified by year of visit. (N=351,558)

**AUROC [95% CI]**					
			**Existing Screening**		
	**2017 (N=63,003)**	**2018 (N=65,368)**	**2019 (N=72,280)**	**2020 (N=76,573)**	**2021 (N=54,364)**
30-Day Suicide Attempt	0.58 [0.54, 0.62]	0.68 [0.62, 0.75]	0.75 [0.71, 0.79]	0.77 [0.72, 0.82]	0.78 [0.71, 0.85]
60-Day Suicide Attempt	0.56 [0.53, 0.59]	0.65 [0.61, 0.69]	0.69 [0.66, 0.72]	0.76 [0.71, 0.80]	0.73 [0.68, 0.78]
90-Day Suicide Attempt	0.56 [0.54, 0.58]	0.63 [0.60, 0.67]	0.69 [0.66, 0.71]	0.74 [0.70, 0.78]	0.73 [0.68, 0.77]
			**Vanderbilt (VU) Model**		
	**2017 (N=63,003)**	**2018 (N=65,368)**	**2019 (N=72,280)**	**2020 (N=76,573)**	**2021 (N=54,364)**
30-Day Suicide Attempt	0.64 [0.59, 0.69]	0.67 [0.60, 0.74]	0.79 [0.75, 0.82]	0.67 [0.62, 0.71]	0.64 [0.58, 0.71]
60-Day Suicide Attempt	0.61 [0.57, 0.65]	0.69 [0.65, 0.73]	0.76 [0.72, 0.78]	0.63 [0.59, 0.68]	0.66 [0.61, 0.71]
90-Day Suicide Attempt	0.65 [0.61, 0.68]	0.67 [0.63, 0.71]	0.75 [0.72, 0.77]	0.65 [0.61, 0.68]	0.68 [0.63, 0.72]
			**MHRN Primary Care Model**		
	**2017 (N=63,003)**	**2018 (N=65,368)**	**2019 (N=72,280)**	**2020 (N=76,573)**	**2021 (N=54,364)**
30-Day Suicide Attempt	0.75 [0.69, 0.80]	0.82 [0.77, 0.87]	0.86 [0.83, 0.90]	0.87 [0.84, 0.90]	0.91 [0.87, 0.94]
60-Day Suicide Attempt	0.77 [0.72, 0.81]	0.79 [0.76, 0.83]	0.84 [0.81, 0.86]	0.84 [0.80, 0.87]	0.87 [0.84, 0.90]
90-Day Suicide Attempt	0.77 [0.74, 0.80]	0.79 [0.75, 0.82]	0.82 [0.79, 0.84]	0.85 [0.81, 0.87]	0.88 [0.85, 0.90]

© 2024 Haroz EE et al. *JAMA Network Open.*

Model Performance by Sex

Finally, given the strong association between outcomes for suicide attempt and death and sex, we examined performance stratified by the patient's sex for suicide attempts. The number of deaths in our dataset was considered too small to conduct a stratified analysis by sex (there were only 4 deaths among females in the 90-day time window that were included in our predictive analysis). Overall, results were similar across both existing screening and both models when stratified by sex. For our main outcome of 90-day suicide attempt, our existing screening procedures, AUROC for was 0.66 [0.64, 0.69] for male patients and 0.66 [0.64, 0.69] for female patients. The VU Model had an AUROC 0.68 [0.66, 0.70] for male patients and 0.68 [0.66, 0.70] for female patients and the MHRN Primary Care model had an AUROC of 0.82 [0.80, 0.84] for male patients and 0.80 [0.80, 0.84] for female patients.

© 2024 Haroz EE et al. *JAMA Network Open.*

eTable 6. Model performance comparing predictions for males and females. (N=351,558)

**AUROC [95% CI]**		
	**Existing Screening**	
	**Male (N=131,135)**	**Female (N=200,452)**
30-Day Suicide Attempt	0.71 [0.67, 0.74]	0.71 [0.67, 0.74]
60-Day Suicide Attempt	0.68 [0.65, 0.70]	0.66 [0.65, 0.70]
90-Day Suicide Attempt	0.66 [0.64, 0.69]	0.66 [0.64, 0.69]
	**Vanderbilt (VU) Model**	
	**Male (N=131,135)**	**Female (N=200,452)**
30-Day Suicide Attempt	0.70 [0.66, 0.73]	0.68 [0.66, 0.73]
60-Day Suicide Attempt	0.68 [0.66, 0.71]	0.66 [0.66, 0.71]
90-Day Suicide Attempt	0.68 [0.66, 0.70]	0.68 [0.66, 0.70]
	**MHRN Primary Care Model**	
	**Male (N=131,135)**	**Female (N=200,452)**
30-Day Suicide Attempt	0.84 [0.82, 0.86]	0.83 [0.82, 0.87]
60-Day Suicide Attempt	0.83 [0.80, 0.84]	0.81 [0.81, 0.85]
90-Day Suicide Attempt	0.82 [0.80, 0.84]	0.80 [0.80, 0.84]

eAppendix 4. Comparison of Demographic Characteristics

We descriptively examined differences in demographic characteristics between the VU and MHRN development samples and the sample included in the current study. There were notable differences in the percentage of patients with a suicide attempt, the proportion of patients on Medicaid, and the proportion of patients who are American Indian or Alaska Native.

© 2024 Haroz EE et al. *JAMA Network Open.*

eTable 7. Comparing demographic characteristics across patient populations used in model development and the current study[a]

	**Current Study**	**MHRN**<br>Visit data only	**Vanderbilt**
N patients	16,835	3,387,741 visits	77, 973
**Demographics**			
Age at first visit	40.2 (17.2)	*NR*	*NR*
Female	8,660 (51.4%)	*2,083,424 (61.5%)*	35,404 (45.4%)
American Indian/Alaska Native[b]	14,251 (84.7%)	37,717 (1.1%)	*NR*
**Outcomes**			
Attempted suicide	324 (1.9%)	8,688 (0.3%)	85 (0.1%)
Death by suicide	37 (0.2%)	445 (<0.1%)	*NR*
**Existing Screening**			
Positive suicide screen	785 (4.7%)	*NR*	*NR*
Positive depression screen	1,697 (10.1%)	*NR*	*NR*
**Insurance**			
Not enrolled	4,868 (28.9%)	*NR*	*NR*
Medicaid	9,398 (55.8%)	160,063 (4.7%)	*NR*
Private	1,858 (11.0%)	2,839,199 (83.8%)	*NR*
Medicare	2,297 (13.6%)	310,001 (9.2%)	*NR*
Other	NR	78,478 (2.3%)	*NR*

© 2024 Haroz EE et al. *JAMA Network Open.*

[a]Categorical variables are summarized as ‘total (percent),’ and continuous variables are summarized as ‘mean (standard deviation).’ Counts less than or equal to 10 (and associated p-values) are suppressed to protect patient privacy.
[b]This is the percentage in the full dataset, but many of the visits by non-AI/AN patients were to clinics excluded from our analytic sample. The analytic sample in which the models were evaluated was 94.0% AI/AN.

© 2024 Haroz EE et al. *JAMA Network Open.*
