# Supplementary material for: Performance of Machine Learning Suicide Risk Models in an American Indian Population
Source: JAMA Netw Open. 2024 Oct 14;7(10):e2439269. doi: 10.1001/jamanetworkopen.2024.39269 (PMC11474420; doi:10.1001/jamanetworkopen.2024.39269)
Supplement: Supplement 2. — Data Sharing Statement [file jamanetwopen-e2439269-s002.pdf]

Data Sharing Statement

Haroz. Performance of Machine Learning Suicide Risk Models in an American Indian Population. *JAMA Netw Open*. Published October 14, 2024.
doi:10.1001/jamanetworkopen.2024.39269

Data
Data available: No

Additional Information
Explanation for why data not available: The Tribe owns the data. The research team are stewards of the data and do not have authority to share it.
